# Clap-and-Fling Mechanism of Climbing-Flight Coccinella Septempunctata

**DOI:** 10.3390/biomimetics9050282

**Published:** 2024-05-09

**Authors:** Lili Yang, Huichao Deng, Kai Hu, Xilun Ding

**Affiliations:** 1 Robotics Institute, Beihang University, Beijing 100191, China; lily_yang@buaa.edu.cn (L.Y.); hukai007@buaa.edu.cn (K.H.); xlding@buaa.edu.cn (X.D.); 2 Beijing Advanced Innovation Center for Biomedical Engineers, Beihang University, Beijing 100191, China

**Keywords:** wing kinematics, climbing flight, “clap-fling”, wing-wing interaction, computational fluid dynamics

## Abstract

Previous studies on the clap–fling mechanism have predominantly focused on the initial downward and forward phases of flight in miniature insects, either during hovering or forward flight. However, this study presents the first comprehensive kinematic data of Coccinella septempunctata during climbing flight. It reveals, for the first time, that a clap-and-fling mechanism occurs during the initial upward and backward phase of the hind wings’ motion. This discovery addresses the previously limited understanding of the clap-and-fling mechanism by demonstrating that, during the clap motion, the leading edges of beetle’s wings come into proximity to form a figure-eight shape before rotating around their trailing edge to open into a “V” shape. By employing numerical solutions to solve Navier–Stokes (N-S) equations, we simulated both single hind wings’ and double hind wings’ aerodynamic conditions. Our findings demonstrate that this fling mechanism not only significantly enhances the lift coefficient by approximately 9.65% but also reduces the drag coefficient by about 1.7%, indicating an extension of the applicability range of this clap-and-fling mechanism beyond minute insect flight. Consequently, these insights into insect flight mechanics deepen our understanding of their biological characteristics and inspire advancements in robotics and biomimetics.

## 1. Introduction

Over the past 30 years, flying insects have garnered significant attention in engineering and science. This interest is due to the similarity in length, velocity scales, and flight conditions between these insects and micro air vehicles (MAVs) [1,2,3]. Micro air vehicles (MAVs) typically feature a wingspan of under roughly 15 cm and weigh less than 100 g, operating at low Reynolds numbers below 10^5^ [4]. During this flow regime, there is a significant deterioration in the aerodynamic performance of fixed wings, particularly in the lift-to-drag ratio and restructure content, while a flapping flight becomes more efficient due to the unsteady flow mechanisms [2,3,5,6,7]. As a result, the lift generation mechanisms associated with the flapping flight of various insects, including fruit flies, bumblebees, hawkmoths, rhinoceros beetles, cicadas, and mosquitoes, have been the subject of extensive research by many scientists [8,9,10,11,12,13,14].

Understanding the aerodynamic characteristics of flapping wings is vital in designing insect-styled micro air vehicles (MAVs). It is exceedingly challenging and practically unfeasible to directly gauge the aerodynamic forces and moments acting on a freely flying insect and to map the flow field around the wings. To overcome the challenge of direct measurement, researchers can employ experimental or numerical methods to estimate the aerodynamic forces and moments on model wing replicas of insect wings [9,15,16,17,18].

**Figure 1:**
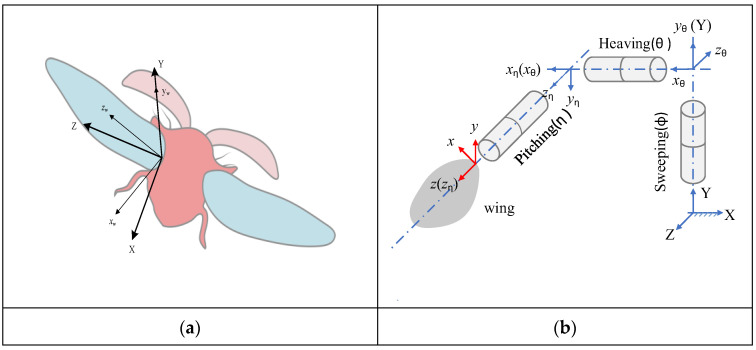
(**a**) Wing kinematics parameters and (**b**) Coordinates.

In the present study, we focus on the Coleoptera beetle species Coccinella septempunctata in Figure 1a, among various flying insects, as it is one of the heaviest of the smaller-size insects and has a pair of thick elytra among the biological flyers that meet the weight and size requirements of MAV [19,20,21,22]. Meanwhile, there have been few investigators who have studied the flight mechanism of the Coleopteran beetle [23,24,25,26,27].

Weis-Fogh initially discovered the clap-and-fling mechanism and its role in enhancing lift while investigating the diminutive wasp Encarsia formosa [28]. Experts typically characterize the clap-and-fling mechanism as such [29,30,31,32]: The clap occurs at the end of the upward wing motion, where the wings’ leading edges make contact. Subsequently, the wings rotate about the leading edge, eliminating the space between them; this action expels a downward jet of fluid due to the clapping motion, resulting in a significant upward force. During the fling phase, which marks the start of the downward wing movement, the wings rotate around the trailing edge, causing them to separate or fling apart rapidly. In the current paper, we designated this mechanism as the “ideal” clap-and-fling mechanism, since no quantitative kinematic measurements were available for the wings to facilitate its detailed description.

Most previous studies have found the clap–fling mechanism in tiny insects, including the greenhouse whitefly Trialeurodes vaporariorum [33], Thrips physique [34], Muscidifurax raptor wasps, and Nasonia vitripennis [35]. The clap-and-fling mechanism, observed in all these species, is thought by researchers to be a pivotal contributor to lift generation in tiny insects, leading to additional inquiries into the unique kinematics of this action [35,36,37,38,39,40]. Reevaluating the mechanism with a focus on the dynamics of wing motion and the generation of force, supported by accurate insect motion data and fluid dynamics analysis, we recommend adjustments to its current portrayal [37]: During the clap phase, the wings adjust to a pronounced angle of attack well before nearing each other, approaching one another with their surfaces nearly in a vertical orientation, followed by an upward vertical movement; in essence, the theoretical depiction of the clap motion diverges significantly from actual observations. The fling phase resembles the theoretical ideal, albeit with an exception. Researchers have studied some novel flap–fling mechanisms [4,36,37,38,41]. In conclusion, because previous research on the flapping mechanism did not involve the Coleoptera, and there is also a lack of research on insect movement other than hovering and forward flight, there has not been a complete understanding of the flapping motion.

The wing system of Coleopteran insects uniquely consists of highly flexible hindwings, which are folded and covered by rigid elytra (forewings). This mechanism differs from that of dragonflies, cicadas, and butterflies, which all have front and rear wing structures. Researchers have not explored small Coleoptera insects’ kinematic measurement and flight mechanisms in climbing flight. The *contributions* are (1) This study presents the first comprehensive kinematic data of the Coccinella septempunctata during climbing flight, revealing a previously unobserved clap-and-fling mechanism during the initial upward and backward phase of the hind wings’ motion. (2) Through numerical simulation of the Navier–Stokes equations, the research demonstrates that the clap-and-fling mechanism significantly enhances lift by approximately 9.65% while reducing drag by about 1.7%, broadening the mechanism’s applicability beyond the flight of minute insects. (3). The findings offer new insights into insect flight mechanics, enhancing our understanding of insect biological characteristics and inspiring advancements in robotics and biomimetics.

## 2. Materials and Methods

### 2.1. Wing Kinematics and Morphological Parameters

We captured Coccinella septempunctata in a school garden. All flight experiments were conducted from 12:00 to 14:00 on the capture day when insects exhibited peak activity levels. Consequently, the researchers selected only the most vigorous individuals as test subjects for the experiments. Due to their positive phototactic behavior, Coccinella septempunctata primarily exhibit climbing flight. In the center of the flight zone, researchers positioned a vertical branch. The initial behavior of Coccinella septempunctata involves flying from the top of this branch, with data collected from the stable phase of its free flight. 

We utilized three orthogonally aligned, synchronized high-speed cameras (i-SPEED 716, capable of capturing 5000 frames per second, with a shutter speed of 50 μs and a resolution of 2048 × 1596 pixels) to capture the 3D kinematics of the wings and body of Coccinella septempunctata during climbing flight. LED arrays, the light source, were employed to illuminate the focal area and enhance image quality. The researchers synchronized the three cameras by inputting the same signal from a signal generator. We developed a specialized toolbox for Matlab designed to extract the positions and orientations of the wings and body of the Coccinella septempunctata from images captured by all three cameras.

Following the flight recording sequence, we subjected the insects to anesthesia and subsequently ascertained the total mass of the Coccinella septempunctata using a BT25S lab scale from Sartorius AG, Göettingen, Germany, with a precision of up to ±0.01 mg. We digitally recorded the configuration of the wings using a microscope that featured an electronic eyepiece boasting a display resolution of 2048 × 1536. The researchers used video sequences to reconstruct the insect’s body shape, treating the cross-sections as simplified elliptical shapes [42]. Once we had collected all morphological parameters, we manually measured body and wing kinematics frame by frame, employing a binocular stereoscopic technique, as suggested in the references [43,44]. This approach required carefully adjusting the models’ position and orientation to achieve an overlay with the physical captures from the three distinct views.

Researchers captured the climbs of a Coccinella septempunctata using high-speed cameras, analyzing wing movements over five wingbeat cycles. Figure 1 illustrates both the movement parameters of the wings and their coordinate system, following established guidelines. This system starts at the left hind wing base, using axes (X, Y, Z) where X is backward, Y is up against gravity, and Z is to the left, with the (X, Z) plane being horizontal. The study outlines how the stroke-plane angle (β) is the angle between this horizontal plane and the path made by the wing’s shoulder and tip during wingbeats. It also sets up a coordinate system (x_s_, y_s_, z_s_), with ys perpendicular to the wing’s movement plane, z_s_ lined up with the *Z*-axis, and xs following a right-hand rule. We then modify this system to match the wing’s movement through three rotations—sweeping, out-of-plane deviation, and pitching—that align with the wing’s thickness, chord, and span directions. The sweeping angle (ϕ) shows the z_s_-axis and *z*-axis relationship on the movement plane. The heaving angle (θ) and the pitching angle (η) measure how the wing tilts and rotates around the *z*-axis.

### 2.2. Aerodynamic Force Computation

In the inertial Cartesian coordinate system, we calculated the aerodynamic forces and flow dynamics surrounding the flapping phase by solving the three-dimensional, unsteady, incompressible Navier–Stokes equations.

(1) ∇⋅u=0

(2) ∂u∂t+u⋅∇u=−∇p+1Re⋅∇2u

Here, u represents the fluid velocity, *t* denotes time, *p* signifies fluid pressure, Re stands for the Reynolds number, ∇ is the gradient operator, and $\nabla^{2}$ is the Laplacian operator.

The wing’s relative velocity and chord length determine the wing’s Reynolds number. Letting U represent the average velocity at the wing’s tip, Re represent the Reynolds number of the wing, and F stand for the aerodynamic force on the wing, the relationship can be expressed as follows:

(3) U=2ΦfR

(4) Re=Ucν

S represents the area of the wing (S = *Rc*). AR is the aspect ratio of the wing (AR = *R*/*c*), and *ν* denotes the kinematic viscosity of the air. Then, numerical simulation was conducted of the beetle flight with air density, *ρ*, 1.225 kg·m^−3^ and dynamic viscosity, μ, 1.87510^−5^ kg·ms^−1^, respectively. The Reynolds number is Re = 1234. 

**Figure 2:**
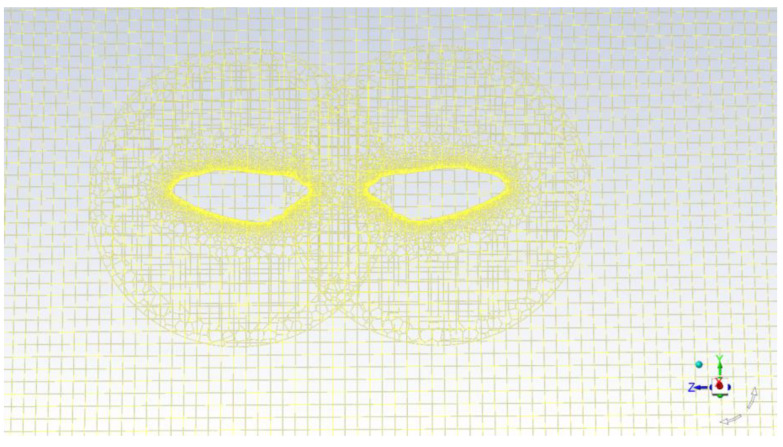
Portions of a computational grid system.

The model’s wings are designed as flat plating with gently curved front and back edges, with the plate’s dimension extending into a thickness of 0.03c. We configured the outline of the model wing in Figure 2 using the wing shape obtained through precise measurement. Due to the relative motion between the left and right hind wings, overlapping moving grids need to be used in the process of solving the equations. A body-aligned curvilinear grid is designated for each wing, complemented by a background Cartesian grid that covers the distant far-field boundary of the flow realm, as shown in Figure 2.

**Figure 3:**
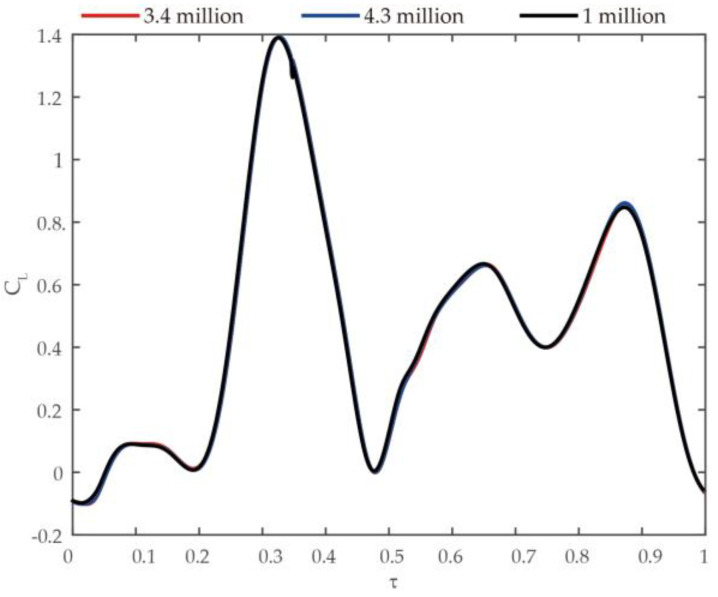
Temporal progression of the lift coefficient (C_L_) for Coccinella septempunctata’s wing in climbing mode over various grid options.

Before exploring the dynamics of flows and aerodynamic forces, we undertook a test to assess grid resolution. We considered three grids: 1 million grids, 3.4 million grids, and 4.3 million. Using the above grids, we performed calculations for the climbing flight of a Coccinella septempunctata; Figure 3 displays the results: It can be seen that as the grid increases, the difference in the lift coefficient of insect flight is within 1%. A finer grid is conducive to the generation of the flow field; hence, we opt for 4.3 million grids.

### 2.3. Morphological Parameters

In Table 1, we summarize the parameters measured for morphology and kinematics. Chord lengths for the hind wing are registered as mean values of $\bar{c}$= 3.5 mm. Similarly, the hind wing has measured wing lengths of *R* = 11.4 mm; here, we state the aspect ratio as AR = *R*/*c*, with aspect ratios AR = 3.257 and masses of single wing = 0.32 mg, respectively. We obtain m = 29.7 mg, and the flapping amplitude equals 190 degrees.

### 2.4. Wing-Flapping Motion at Climbing Flight

**Figure 4:**
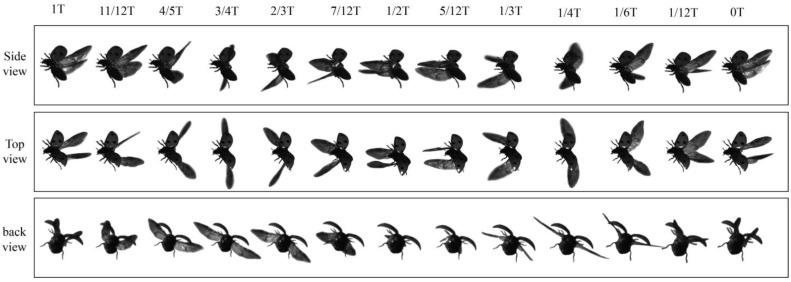
The videos of Coccinella septempunctata in climbing motion, featuring perspectives from three cameras. The time notations represent microseconds starting from the downstroke’s commencement.

Experts often describe the clap-and-fling mechanism: In the clap phase, the leading edges of the insect’s wings come together as the upstroke concludes. Subsequently, the wings pivot around this leading edge, eliminating the space between them. In the fling phase, the wings open up by rotating around the trailing edge at the start of the ensuing downstroke. The clap–fling mechanism occurs at the end of the upstroke or the beginning of the downstroke. During our study, we successfully recorded the free-climbing flight patterns of Coccinella septempunctata. The movies in Figure 3 (Multimedia view) give the video sequences for one cycle. For example, Figure 4, at t = 0, shows the sequences of one of the Coccinella septempunctata’s flights, marking the beginning of the downstroke; it is observable from the movement sequence in the figure that a clap–fling mechanism is present at the end of the downstroke or the beginning of the upstroke as well. This clap–fling motion concludes at the end of the downstroke when the wings approach each other near the leading edge. As the upstroke initiates, the wings unfold around the trailing edge, forming a V-shape. The clap–fling mechanism is present during the upstroke and the downstroke phase.

## 3. Results and Discussion

**Figure 5:**
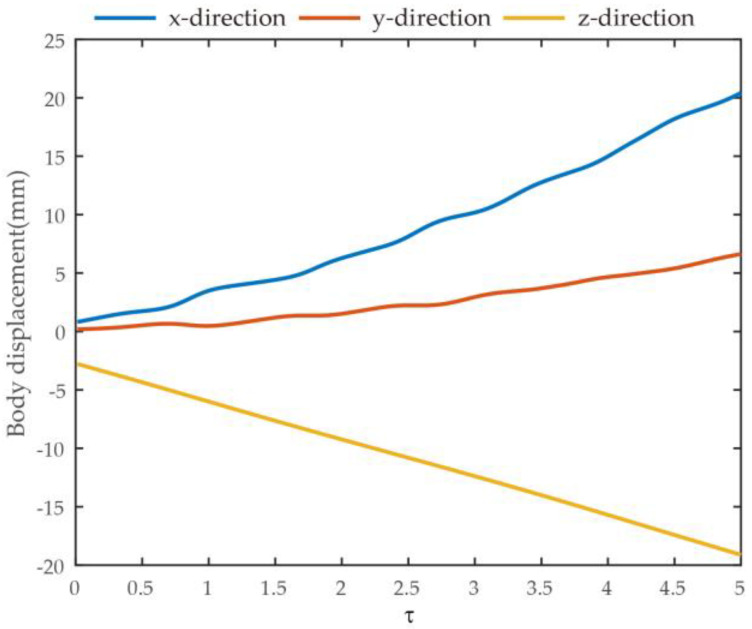
The measured body-translational kinematics of Coccinella septempunctata in climbing flight.

In the climbing flight of the Coccinella septempunctata, if the displacement upwards (*y*-axis) and forwards (*x*-axis) is approximately three times the lateral displacement (*z*-axis), this indicates that the Coccinella septempunctata primarily focuses on vertical and forward movement, as shown in Figure 5. It achieves more efficient climbing and forward flight by increasing the amplitude and frequency of wing flapping, generating more significant lift and propulsive force.

The pitch angle η is related to the angle of attack of the wing (α) as follows: α = η in the upstroke and α = 180° − η in the downstroke. Measurements taken included the wingbeat frequency (n) and stroke-plane angle (β), as well as the body angle (χ)—the angular displacement of the long axis of the insect’s body to the horizontal. β + χ is the angle between the stroke plane and the long axis of the insect body. The notation β represents the angle between the stroke plane and the body’s long axis, which is 61.01°. A non-dimensional time parameter, denoted as (τ), has been defined for clarity in data representation: (τ) starts at 0 at the beginning of a downstroke and reaches 1 by the end of the succeeding upstroke.

**Figure 6:**
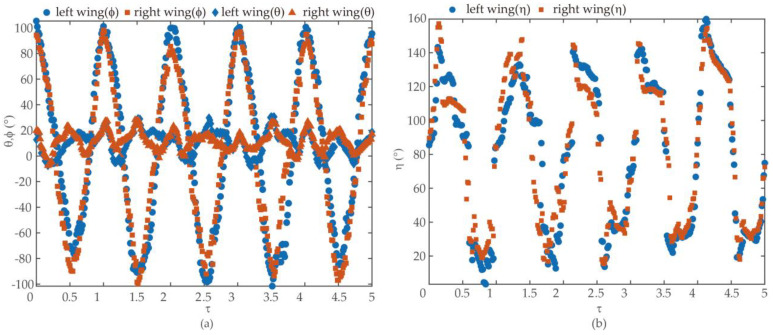
Wing kinematics of Coccinella septempunctata (**a**) Instantaneous wing kinematics of Coccinella septempunctata ϕ, sweeping angle; θ, deviation angle; (**b**) η pitch angle.

**Figure 7:**
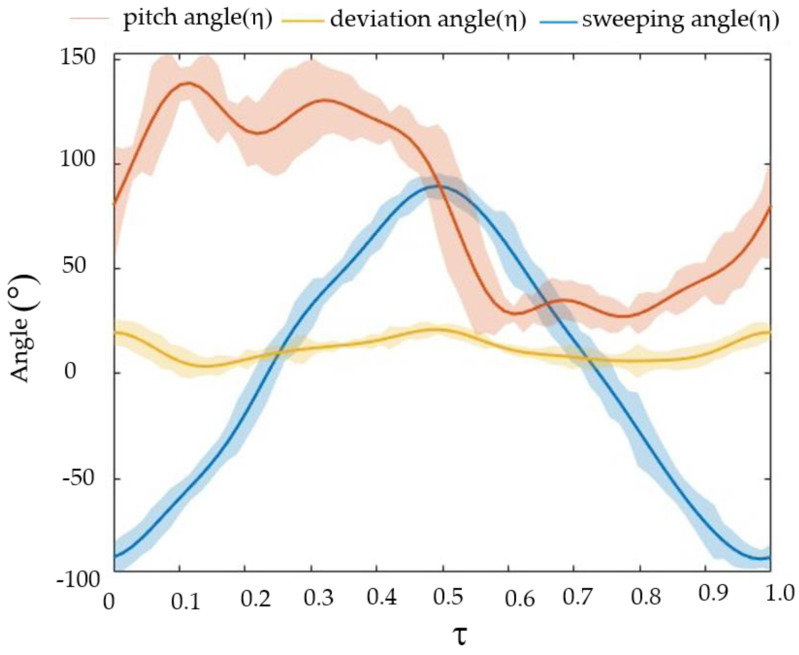
The kinematics of the hind wing and the shaded region represent the instantaneous standard deviation.

Figure 6 shows the parameters of wing kinematics of the hind wing in five cycles. We created the graph with the data value averaged from three extractions using the DLT method in Figure 7. The shaded area represents the immediate standard deviation. There were 53 frames for a single stroke, meaning the frequency f was 72.5 Hz ± 0.3 Hz. The recording indicated a flapping angle of 190 degrees. The stroke angle for the hind wing stood at 61.03 degrees, subject to a variance of 2.3 degrees. Deviations remained confined below 20 degrees. We observed a much broader spectrum for the angle of attack (AOA), which varied from 5 degrees to a space of 160 degrees—for the hind wing, the flapping periods exhibited asymmetry between downstroke and upstroke. We approximated the angle values for each wing as 8th-order Fourier series:

(5) $$\varphi \left(t\right)=\sum_{n=0}^{n=8}\left[\varphi_{cn}\cos\left(nkt\right)+\varphi_{sn}\sin\left(nkt\right)\right]$$

(6) $$\alpha \left(t\right)=\sum_{n=0}^{n=8}\left[\alpha_{cn}\cos\left(nkt\right)+\alpha_{sn}\sin\left(nkt\right)\right]$$

(7) $$\beta \left(t\right)=\sum_{n=0}^{n=8}\left[\beta_{cn}\cos\left(nkt\right)+\beta_{sn}\sin\left(nkt\right)\right]$$

We derived the coefficients $\varphi_{cn}$, $\varphi_{sn}$, $\beta_{cn}$, $\beta_{sn}$, $\alpha_{cn}$, and $\alpha_{sn}$ by solving a matrix equation, Equations (3.1)–(3.3) in the reference [23], using experimental data and then used them as input to describe the motion of the wing surface in the flow solver.

Figure 7 (the curves) presents the filtered values of ϕ, θ, and η as functions of τ. In a wingbeat cycle, the researchers took approximately 69 pictures. They then determined a linear regression line of the projections. The definition of a stroke plane is a plane that is parallel to the above line and passes through the wing base.

### The Aerodynamic Forces on the Wings

The resolution of the incompressible Navier–Stokes equations, detailed in Section 2, determines the aerodynamic forces acting on the insect and the surrounding fluid flows. In the ensuing discourse, we will use the right wing as a representative example, given that both wings mirror the aerodynamic force and flow-field structure during the fling motion.

**Figure 8:**
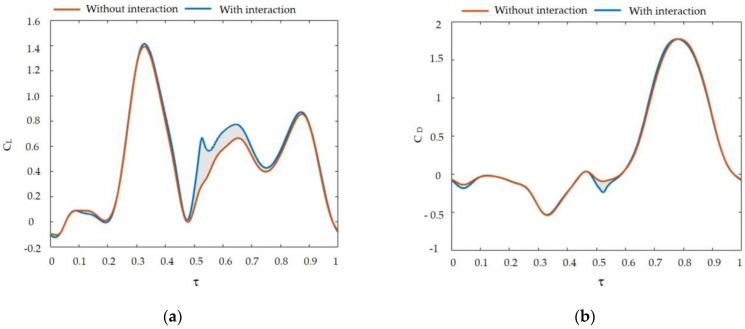
(**a**) Time courses of the computed C_L_; (**b**) C_D_ of the right wing with and without interaction.

We can define the lift and drag of the wing as the components of the total aerodynamic force that act perpendicular and parallel to the wing’s relative velocity at the radius of gyration (r_2_) of the wing. A wing’s vertical and horizontal forces are attributed to its lift and drag. Now, we will examine the generation of lift and drag by analyzing the lift (L) and drag (D) of the wings of Coccinella septempunctata throughout the flapping cycle. Refer to Figure 8 for a visualization of these quantities.

As observed from the temporal profiles of C_L_ and C_D_ in Figure 8, the lift force produced in the case of real double-wing flapping is always better than the result of single-wing flapping. The main contribution to the mean lift originates from the middle sections of the upstroke and downstroke phases (Notice the prominent peaks in C_L_ occurring around τ ≈ 0.2–0.48 and τ ≈ 0.78–0.9, as depicted in Figure 8a). Based on the data in Figure 8a, researchers estimate that the middle sections of the upstroke and downstroke phases contribute approximately 66% of the mean lift. The opening phase of the wings produced 9.65% of the lift while concurrently reducing drag by about 1.7% in Figure 8b.

Table 2 shows the single and double wings under the same parameters and the resulting hind-wing lift and drag coefficients over time with and without interaction. It calculates the total sum of lift and drag coefficients over a cycle. The opening phase of the wings produced 9.65% of the lift while concurrently reducing drag by about 1.7%.

**Figure 9:**
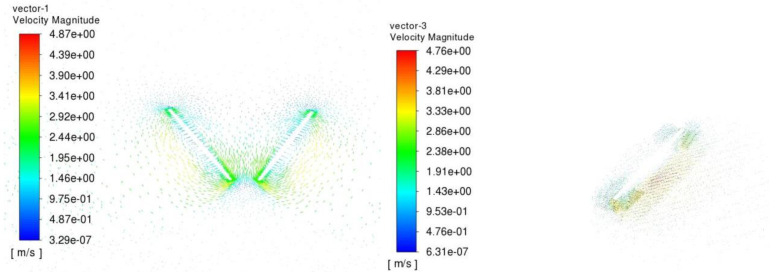
The velocityvector plots are displayed in a vertical plane 6.2027R from the wing for both the double and single wing during the fling.

Observations indicate that the leading-edge vortex resulting from wing–wing interaction is significantly more robust, though to a moderate extent, than that produced by a single wing alone. This suggests that wing interactions create a more robust “downward-velocity jet”, exceeding the effect seen with just one wing. Consequently, this results in the production of a greater vertical force. Indeed, examining Figure 9 confirms this phenomenon, as it depicts the velocity-vector plots in a vertical plane at a distance of 0.62027R from the wing root. Hind wings with wing–wing interaction produce a high-speed, almost downward jet, in contrast to the weaker downward flow generated by the single wing.

**Figure 10:**
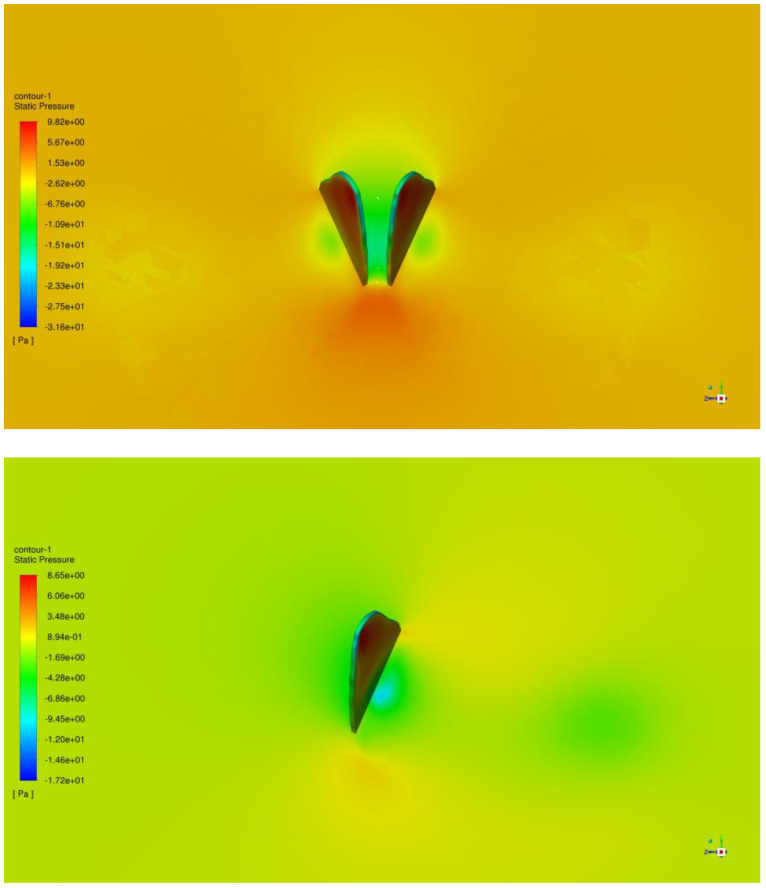
Upper-surface pressure differences between the right wing with and without interaction.

We then look at the fling phase (t/T = 0.55–0.65), plotting the pressure data in Figure 10. The wings undergo rapid rotation around their trailing edges during the fling phase, starting from zero rotational velocity. This rotation generates significant and opposing vorticity at various locations. Furthermore, the wings forcefully strike the fluid during the fling phase, generating a substantial impulse. This action leads to a pronounced positive pressure on the lower surface of the wings and a notable suction pressure on the upper surface. Thus, the interaction between the wings has a positive effect on lift.

## 4. Conclusions and Future Work

This study has significantly expanded the knowledge of the clap-and-fling mechanism in insect flight, particularly beyond the previously studied scope of miniature insects during hovering or forward flight. Through the comprehensive kinematic analysis of Coccinella septempunctata during its climbing flight, we unveiled a novel phase of the clap-and-fling mechanism occurring during the hind wings’ initial upward and backward motion. This phase is characterized by the wings’ leading edges coming into proximity to form a figure-eight shape, subsequently rotating around their trailing edge into a “V” shape to open. Such a mechanism was previously undocumented in giant insects like Coccinella septempunctata, marking a groundbreaking discovery in insect flight dynamics.

Utilizing numerical solutions for solving Navier–Stokes equations enabled a detailed simulation of the aerodynamic conditions influencing single and double hind wings. Our findings conclusively demonstrated that the opening phase of the clap-and-fling mechanism enhances lift by approximately 9.65% while concurrently reducing drag by about 1.7%. We estimate that the middle sections of the upstroke and downstroke phases contributed approximately 66% of the mean lift. This finding broadens the applicability of the clap-and-fling mechanism, indicating its relevance for the flight of tiny insects and larger species.

Consequently, this research deepens the scientific community’s understanding of insect flight mechanics and illuminates potential pathways for innovation in robotics and biomimetics. By demonstrating the aerodynamic efficiency of the clap-and-fling mechanism in a larger insect species, we pave the way for developing more efficient biomimetic flying robots, potentially revolutionizing current designs by leveraging the natural efficiency of insect flight dynamics. Future studies should continue to explore the application of these findings in designing advanced robotic systems that mimic these biological flight mechanisms, potentially leading to breakthroughs in aeronautics and engineering.

In the future, we plan to conduct comparative studies of the clap-and-fling mechanism among various beetle species and other insects. This approach will clarify whether the kinematic patterns and aerodynamic advantages we observed in Coccinella septempunctata are unique to this species or common among others. Such research could enhance our understanding of the evolution of flight mechanics.

## Figures and Tables

**Table 1:** Morphological parameters and kinematic parameters of the Coccinella septempunctata.

Parameters	Hind Wing
Sweeping amplitude (*Φ*)	190°
Mean chord length ($\bar{c}$)	3.5 mm
Wing length (*R*)	11.4 mm
Aspect ratio (AR = *R*/$\bar{c}$)	3.257
Mass (*m*)	0.00032 g
Frequency (*f*)	72.5
Total mass (including legs)	0.0297 g
Mean tip velocity of the hind wing (*U_h_*)	5.287 m/s

**Table 2:** Morphological parameters and kinematic parameters of the Coccinella septempunctata.

Parameters	Hind Wing (with Interaction)	Hind Wing (without Interaction)
Sweeping amplitude (Φ)	190°	190°
Mean chord length ($\bar{c}$)	3.5 mm	3.5 mm
Wing length (*R*)	11.4 mm	11.4 mm
Frequency (*f*)	72.5	72.5
Mean tip velocity of the hind wing (*U_h_*)	5.287 m/s	5.287 m/s
Total lift coefficient	0.497103	0.453338
drag coefficient	0.280235	0.285165

## Data Availability

The datasets used and analyzed during the current study are available from the corresponding author upon reasonable request.
